# P-2050. Enhancing Patient Outcomes and System Efficiency Through Active Screening for Recurrent Clostridioides difficile and Specialty Treatment at an Infectious Diseases Clinic

**DOI:** 10.1093/ofid/ofaf695.2214

**Published:** 2026-01-11

**Authors:** Thomas Holowka, Trieu-Vi Khuu, Nikolaos Mavrogiorgos, Elizabeth C Arant, Michael Herce, Claire E Farel, Sarah McGill, Luther A Bartelt

**Affiliations:** Stony Brook University, Stony Brook, NY; University of North Carolina, Chapel Hill, North Carolina; University of North Carolina Medical Center, Chapel Hill, North Carolina; University of North Carolina at Chapel Hill, Chapel Hill, NC; University of North Carolina, Chapel Hill, North Carolina; UNC Chapel Hill, Chapel Hill, North Carolina; University of North Carolina, Chapel Hill, North Carolina; University of North Carolina School of Medicine, Chapel Hill, NC

## Abstract

**Background:**

Recurrent *Clostridioides difficile* infection (rCDI) increases healthcare costs and leads to poor patient outcomes. FDA-approved fecal microbiota transplant (FMT) therapies that are effective at breaking the cycle of recurrence are now available in the ambulatory setting without a need for colonoscopic delivery. To adapt to these changes, starting in 6/2024 we piloted routing of outpatient rCDI referrals to the Infectious Diseases (ID) clinic instead of Gastroenterology (GI). We coupled this shift in clinical care with an active rCDI screening process with the overall goal of improved access to care in a large academic medical system.

**Figure 1:**
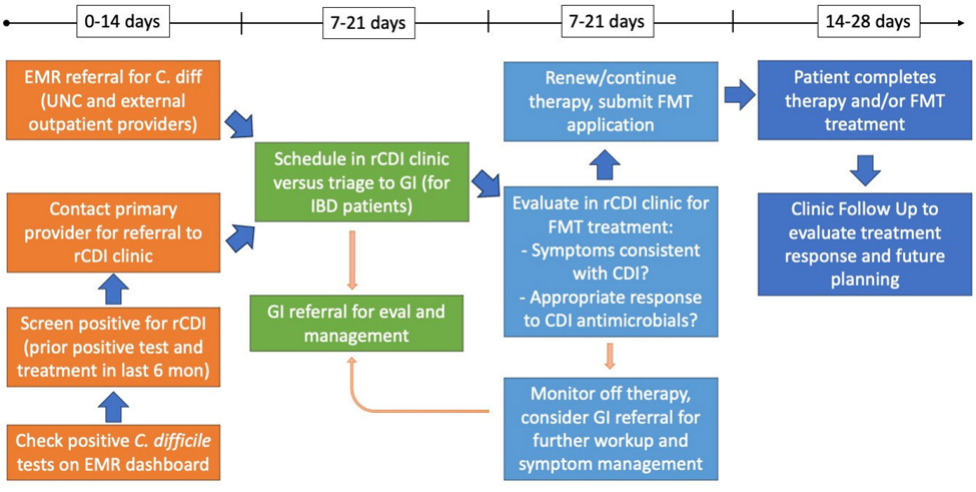
Active Screening Process and Treatment Algorithm for rCDI clinic. Screening and referral process in orange, scheduling and triage in green, clinic evaluation and treatment in light blue, follow up in dark blue, with target timeline above. EMR = Electronic Medical Record, IBD = Inflammatory Bowel Disease, FMT = Fecal Microbiota Transplant.

**Table 1:**
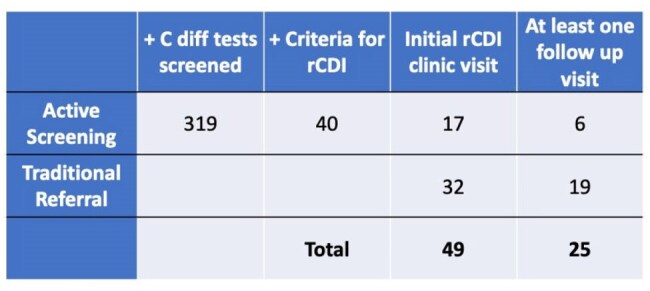
rCDI referrals and patients seen in ID clinic Includes all patients referred for rCDI post-6/2024 to ID clinic through active screening or traditional provider-driven referral pathway.

**Methods:**

An active screening approach was used to identify patients through the EMR with positive *C. difficile* testing in real time who qualified as having rCDI (prior positive test with treatment within 6 months) (Figure 1). Data on time from positive test to referral and to initial visit was compiled as an access metric. Student’s T-test was used for statistical comparison of time to care between patients referred through active screening to those referred through traditional provider-driven pathways and for patients referred to GI clinic pre-6/2024.

**Table 2:**
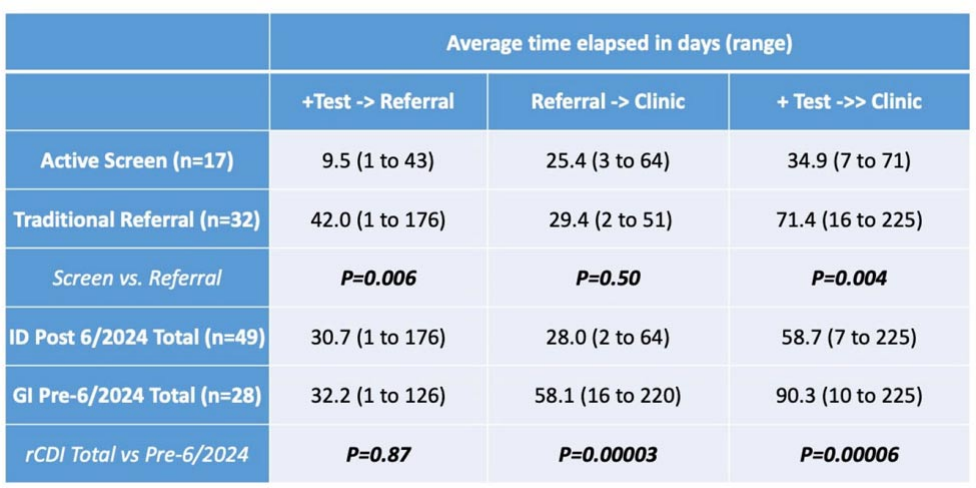
Test to referral to clinic time Comparisons of time from positive C. difficile test to referral and to initial clinic visit. Student’s T-test performed for all comparisons.

**Figure 2:**
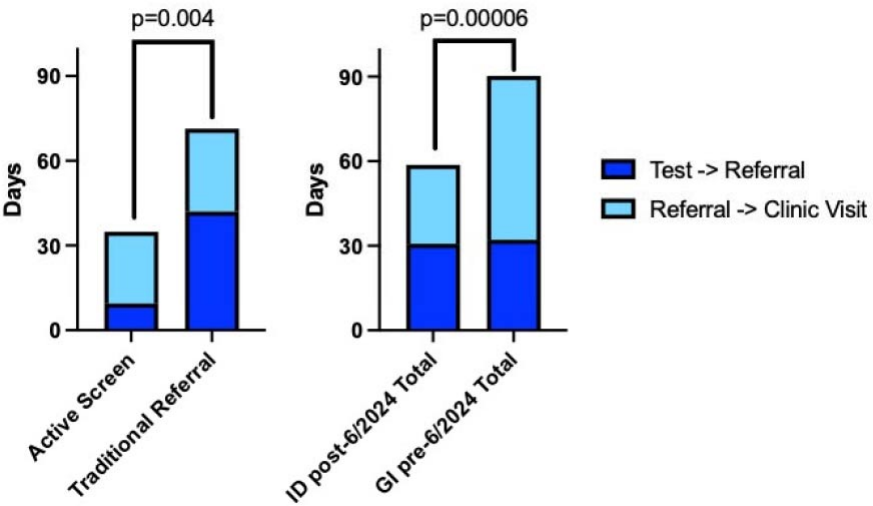
Test to referral to clinic time comparison Comparisons of time from positive C. difficile test to referral and to initial clinic visit. Average time depicted with comparison of time from positive test to initial clinic visit using Student’s T test.

**Results:**

From 6/2024 to 4/2025, 319 positive *C. difficile* tests were identified yielding 40 patients with rCDI, 17 of whom were seen in ID clinic. In addition, 32 patients referred through traditional pathways were seen yielding 49 total new rCDI visits in ID clinic (Table 1). For traditional vs. active screening referrals, time from last positive *C. difficile* test to referral was reduced from 42.0 to 9.5 days and time from last positive test to clinic visit was reduced from 71.4 to 34.9 days. For referrals to GI pre-6/2024 vs. those to ID post-6/2024, time from referral to clinic visit was reduced from 58.1 to 28.0 days, and time from positive test to clinic visit from 90.3 to 58.7 days (Table 2, Figure 2).

**Conclusion:**

Time to access rCDI care was reduced by a month after transitioning referrals to ID clinic, with further reduction by a month in patients identified through an active screening approach. These improvements enhance patient safety and satisfaction by ensuring quicker access to care with reduced wait times, and lower healthcare costs by streamlining the referral process.

**Disclosures:**

Sarah McGill, MD, MSc, EvoEndo: Grant/Research Support|Revivicor: Grant/Research Support Luther A. Bartelt, MD, NIH: Grant/Research Support

